# Low Dose Oral Alitretinoin With Narrowband Ultraviolet B Therapy for Chronic Hand Dermatitis

**DOI:** 10.1177/12034754211071123

**Published:** 2022-01-23

**Authors:** E. Dimitra Bednar, Mohannad Abu-Hilal

**Affiliations:** 1 12362 Michael G. DeGroote School of Medicine, McMaster University, ON, Canada; 2 152997 Division of Dermatology, Faculty of Health Sciences, McMaster University, ON, Canada

**Keywords:** alitretinoin, chronic hand dermatitis, dyshidrotic eczema, narrowband ultraviolet B, phototherapy

## Abstract

**Background:**

Chronic hand dermatitis (CHD) is difficult to treat and has high individual and societal burdens. Phototherapy and oral alitretinoin are safe monotherapies for CHD, but their combination has not been assessed.

**Objective:**

To assess the effectiveness and safety of low dose oral alitretinoin combined with phototherapy versus high dose oral alitretinoin for CHD refractory to topical corticosteroids.

**Methods:**

This retrospective study of adult patients with CHD refractory to topical corticosteroid therapy compared low dose oral alitretinoin (10 mg three times weekly) combined with narrowband ultraviolet B therapy (three times weekly; LDA-UVB) to high dose oral alitretinoin (30 mg daily; HDA) for 16 weeks. Outcomes were improvement in disease severity measured by the Physician’s Global Assessment and quality of life measured with the Dermatology Life Quality Index.

**Results:**

The mean age of the study population (*n* = 64) was 41.25 years and 57.8% were male. Both cohorts experienced improvements in disease severity and quality of life after 16 weeks, however, significantly more participants who received LDA-UVB (*n* = 21/33, 63.6%) achieved “clear” or “almost clear” assessments compared to those who received HDA (*n* = 12/31, 38.7%; *P* < .05). Adverse effects were significantly more prevalent in the HDA group (*P* < .0001) and included headache, elevated cholesterol, and dry lips.

**Conclusions:**

The combination of low dose oral alitretinoin with narrowband-UVB therapy was more effective and had fewer adverse effects compared to high dose oral alitretinoin for participants with CHD refractory to topical corticosteroid therapy.

## Introduction

Chronic hand dermatitis (CHD) is an inflammatory non-infectious eruption of the hands that persists for more than 3 months or has at least two relapses within one calendar year.^1^ CHD can be classified by its etiology or by its morphology into the following subtypes: hyperkeratotic hand dermatitis (HHD), dyshidrotic hand eczema (DHE), and fingertip dermatitis (FTD).^2,3^ The prevalence of hand dermatitis is approximately 10% in the general population and it is estimated that between 5% to 10% of patients with hand dermatitis will develop CHD.^4,5^ People with CHD have reduced quality of life (QOL), impaired psychosocial wellbeing, and increased absences from work.^6,7^ The disease burden translates to society with associated healthcare costs and lost productivity.

The approach to treatment of CHD is stepwise and begins with moisturizers and emollients, avoidance of allergens and irritants, and application of moderate- to high-potency topical corticosteroids.^1^ Approximately 50% to 60% of patients with CHD will not respond to these treatments.^8-10^ There are limited options for CHD refractory to topical corticosteroids. Alitretinoin (9-cis retinoic acid) is an endogenous retinoid that was approved for the treatment of severe CHD that does not respond adequately to potent topical corticosteroids by the European Medicines Agency in 2008 and by Health Canada in 2010.^11,12^ Oral alitretinoin received approval after a randomized double-blind placebo-controlled multicentre phase-III trial (BACH) in Canada and Europe of n = 1,032 patients with severe refractory CHD demonstrated its efficacy and safety.^2^ Forty-eight percent of participants treated with 30 mg of oral alitretinoin once daily for 24 weeks achieved Physician Global Assessment (PGA) scores of “clear” or “almost clear” compared to 17% who received placebo (*P* < .001).^2^ Another pivotal multicentre clinical trial (HANDEL) confirmed oral alitretinoin was effective in reducing the severity of CHD.^3^ Treatment with alitretinoin was associated with a significantly faster time to response than placebo (*P* < .001) and had a significantly positive effect on QOL.^3^ Evidence from real-world use is consistent with these findings.^12-14^ Common adverse effects occurring in >5% of patients include headache, increased cholesterol and triglyceride levels, mucocutaneous events, and decreased thyroid-stimulating hormone (TSH). These effects are dose-dependent and occur more frequently in participants who receive higher doses.^2,15^ Between 5% and 10% of participants in the literature have withdrawn from treatment due to adverse effects or clinically relevant changes of laboratory values.^2,3,12,15^

Phototherapy is an alternative treatment for CHD refractory to topical corticosteroids.^1,16^ Controlled trials of ultraviolet A (UVA), psoralen-plus UVA (PUVA), UVB, and narrowband-UVB (NB-UVB) for the treatment of CHD determined that NB-UVB had the fewest side effects and comparable efficacy.^17-20^ Unfortunately, phototherapy of any kind is only modestly effective, with response rates ranging from 8% to 40%.^21-23^

The evidence to treat CHD refractory to topical corticosteroid therapy is inadequate and limited.^23^ The combination of oral retinoids with ultraviolet therapy (Re-UV) has been available as a treatment modality for other cutaneous conditions for almost half a century. Despite evidence in psoriasis and cutaneous T-cell lymphoma, Re-UV remains an underutilized therapeutic modality.^24^ In both of these conditions, Re-UV was found to be more effective and had fewer adverse effects than either monotherapy. The objective of this retrospective cohort study was to assess the effectiveness and safety of low dose oral alitretinoin (10 mg three times weekly) in combination with narrowband ultraviolet B therapy (three times weekly; LDA-UVB) relative to high dose alitretinoin (30 mg daily; HDA) administered for 16 weeks for the treatment of CHD refractory to topical corticosteroids.

## Methods & Patients

### Study Design

This study was approved by the institutional review board. A retrospective review of electronic medical records was conducted for adult patients (>18 years of age) with CHD refractory to topical corticosteroids and treated with LDA-UVB or HDA from 2017 to 2020.

### Participants

Patients with HHD, DHE, and FTD were included. Eligible participants were required to i) meet formal criteria for CHD (persistent for at least 3 months, or with two or more flares within one calendar year); ii) have tried potent topical corticosteroids (clobetasol propionate 0.05% ointment daily for 3 weeks, then 1 week off) for at least 8 weeks; and iii) have failed to respond or were unable to achieve remission with topical corticosteroids.

### Interventions

The HDA group received the recommended dose of 30 mg of oral alitretinoin daily. The LDA-UVB group received 10 mg of oral alitretinoin on Mondays, Wednesdays and Fridays, with NB-UVB therapy delivered on the same days. Participants were started on oral alitretinoin and NB-UVB simultaneously. The initial starting dose of NB-UVB was 75% of the recommended dose based on Fitzpatrick skin type. All participants provided informed consent after a thorough discussion of the risks and benefits associated with either treatment. Female participants of child-bearing age were required to start two methods of contraception. Treatment duration was 16 weeks for both cohorts with follow up every 4 weeks. Participants were instructed to use only moisturizers concurrently.

### Outcome Measures and Statistical Analyses

The primary outcome was response to treatment, defined as achieving a rating of “clear” or “almost clear” (scores of 0 or 1, respectively) on the PGA after 16 weeks of treatment. The PGA is a measure of disease severity scored on a Likert scale from zero to four. A PGA score of 2 indicates mild disease, a score of 3 indicates moderate disease, and a score of 4 indicates severe disease. The secondary outcome was an improvement in QOL, defined as an at least 50% reduction in the Dermatology Life Quality Index (DLQI) after 16 weeks of treatment. The DLQI is a patient-reported measure of the impact their skin condition has on QOL. The index is scored from zero to 30 and higher scores indicate a greater burden of disease; scores between 0-1 indicate no impact, scores 2-5 indicate a small impact, scores 6-10 indicate a moderate impact, scores 11-20 indicate a very large impact, and scores 21-30 indicate an extremely large impact. The proportion of participants who experienced an improvement in their QOL and the percent reduction in DLQI scores were measured for both treatment groups. Adverse effects were recorded at each follow up appointment. Laboratory investigations were completed monthly and included a complete blood count (CBC), lipid profile including total cholesterol and triglyceride levels, ALT, TSH, bilirubin, and β -HCG for female participants of childbearing age.

Wilcoxon’s rank sum test was used to compare changes in PGA and DLQI scores within and between groups. Fisher’s exact test was used to compare the frequency of adverse effects between the two treatment groups. All statistical analyses were conducted in RStudio 1.4 (Boston, MA) using one-tailed *p*-values with significance determined *a priori* at ∝ <.05.

## Results

A total of n = 64 participants were included with a mean age of 41.25 years (range, 22-62 years). More than half (57.8%) of the participants were male. The most common skin type was Fitzpatrick 2 (*n* = 30/64, 46.9%). Mean duration of disease before study inception was 3.14 years (range, 1-9 years). The majority of participants had HHD (*n* = 45/64, 70.3%), followed by DHE (*n* = 14/64, 21.9%) and FTD (*n* = 5/64, 7.8%). Just 18.8% (n = 12/64) of participants had undergone patch-testing and 12.5% (*n* = 8/64) had a previous skin biopsy.

Thirty-three participants received LDA-UVB and thirty-one participants received HDA. Baseline demographics and clinical characteristics of the cohorts were comparable and are summarized in Table 1.

**Table 1:** Baseline Demographics and Characteristics of the Study Population.

Variable	ALL (*n* = 64)	LDA-UVB (*n* = 33)	HDA (*n* = 31)
Mean age (range)	41.25 (22-62)	42.03 (22-62)	40.42 (24-58)
% Male	57.8%	54.5%	61.3%
Fitzpatrick Skin Types			
1	8 (12.5%)	6 (18.2%)	2 (6.5%)
2	30 (46.9%)	11 (33.3%)	19 (61.3%)
3	14 (21.9%)	9 (27.3%)	5 (16.1%)
4	8 (12.5%)	4 (12.1%)	4 (12.9%)
5	4 (6.2%)	3 (9.1%)	1 (3.2%)
Diagnoses			
HHD	45 (70.3%)	23(69.7%)	22 (71.0%)
DHE	14 (21.9%)	7 (21.2%)	7 (22.6%)
FTD	5 (7.8%)	3 (9.1%)	2 (6.4%)
Mean duration of disease in years (range)	3.14 (1-9)	3.03 (1-7)	3.26 (1-9)
Initial PGA scores (%)			
3	20 (31.3%)	13 (39.4%)	7 (22.6%)
4	44(68.7%)	20 (60.6%)	24 (77.4%)
Median initial DLQI scores (range)	13 (7, 23)	13 (7, 23)	14 (9, 23)

Abbreviations: DHE: dyshidrotic hand eczema; DLQI: dermatology life quality index; FTD: fingertip dermatitis; HDA: high dose alitretinoin; HHD: hyperkeratotic hand dermatitis; LDA-UVB: low dose alitretinoin with narrow band UVB therapy; PGA: physician global assessment.

Both cohorts had statistically significant improvements in disease severity from baseline after 16 weeks (**Table 2**). The initial mean PGA score for the LDA-UVB cohort was 3.61 (range, 3-4), and the final PGA score was 1.36 (range, 0-4) (*P* < .0001). Among the HDA cohort, the initial mean PGA score was 3.77 (range 3-4) and the final mean PGA score was 1.77 (range 0-4), (*P* < .0001). Significantly more participants in the LDA-UVB group (*n* = 21/33, 63.6%) achieved a PGA rating of “clear” or “almost clear” hands compared to the HDA group after 16 weeks (*n* = 12/31, 38.7%; *P* < .05).

**Table 2:** Results at 16 weeks.

Variable	LDA-UVB (n = 33)	HDA cohort (n = 31)	*P* value
Mean final PGA score (range)	1.36 (0, 4)	1.77 (range 0-4)	0.09
Final PGA scores of 0-1 (%)	21 (63.6%)	12 (38.7%)	0.04
Median final DLQI score (range)	7 (1, 11)	8 (2, 15)	0.01
Number (%) reporting >50% reduction in final DLQI score	15 (45.4%)	12 (48.4%)	0.11
Adverse effect of any type (%)	13 (39.4%)	23 (74.2%)	4.8 × 10^–5^
Headache (%)	2 (6.1%)	11 (35.5%)	4.0 × 10^–3^
Elevated cholesterol (%)	2 (6.1%)	8 (25.8%)	0.03
Dry lips (%)	2 (6.1%)	8 (25.8%)	0.03

Abbreviations: DLQI, dermatology life quality index; HDA, high dose alitretinoin; LDA-UVB , low-dose alitretinoin with narrowband UVB therapy; PGA, physician’s global assessment.

Wilcoxon rank sum test was used to compare continuous or nominal variables and Fisher’s exact test used to compare categorical data between the LDA-UVB and HDA groups.

Both cohorts reported statistically significant improvements in their QOL at 16 weeks (Table 2). The initial median DLQI score for the LDA-UVB cohort was 13 (range, 7-23), and the final medial DLQI score was 7 (range, 1-11) (*P* < .0001). The HDA cohort reported an initial median DLQI score of 14 (range, 9-23), and a final median DLQI score of 8 (range, 2-15) (*P* < .0001). The proportion of participants who experienced an improvement in their QOL (50% or greater reduction in their final DLQI score) at 16 weeks was comparable between cohorts; 45.4% (*n* = 15/33) achieved this outcome in the LDA-UVB group compared to 48.4% (*n* = 15/31) in the HDA group (*P* > .05). The proportion of participants who reported their CHD had either “no” or a “small” impact on their QOL at 16 weeks was similar between cohorts (LDA-UVB *n* = 15/33, 45.4%; HDA *n* = 11/31, 35.4%, *P* > .05).

Adverse effects of any type were significantly more prevalent in the HDA cohort compared to the LDA-UVB cohort (*P* < .0001). Within the HDA cohort, 74.2% (*n* = 23/31) experienced at least one adverse effect compared to 39.4% (*n* = 13/33) of the LDA-UVB cohort. Analysis of individual adverse effects revealed significantly more participants in the HDA group had headaches (*n* = 11/31,35.5%), elevated cholesterol (*n* = 8/31, 25.8%), and dry lips (*n* = 8/31, 25.8%) compared to the LDA-UVB group. No participants in either cohort discontinued treatment due to adverse effects and no participants experienced serious adverse medical events. The combination of low dose oral alitretinoin with NB-UVB therapy was well tolerated in this studied cohort.

## Discussion

This study compared LDA-UVB to HDA for patients with CHD refractory to topical corticosteroids in a real-world setting. Participants who received LDA-UVB for 16 weeks had significantly better outcomes in skin clearance and fewer adverse effects compared to participants who received HDA. These findings suggest that the combination of low dose oral alitretinoin with phototherapy is well tolerated and may produce better results with fewer adverse effects than the recommended higher doses of oral alitretinoin alone for CHD.

Re-UV has been a treatment option for moderate to severe plaque psoriasis for almost 4 decades^24^ and has demonstrated superior results than either monotherapy in treating psoriasis and cutaneous T-cell lymphoma (CTCL).^24-33^ Several double-blind randomized controlled trials determined that Re-UV combination therapy was more effective than placebo-UV or UV alone at clearing psoriasis.^26-30^ Participants who received Re-UV had significantly greater response rates and lower cumulative UV energies.^27,29^ These pivotal studies confirmed the synergistic effects of Re-UV. Retinoids thin the stratum corneum and enhance UV penetration, making the skin more susceptible to UV radiation and inducing a faster response.^34^ Thus, lower cumulative dosages of UV light may be effective, and the long-term carcinogenic risk of UV treatment may be reduced. Meanwhile, concurrent phototherapy permits lower dosages of retinoids to be effective and minimizes their dose-dependent adverse effects.^27,29^ Studies of Re-UV combination in patients with CTCL found it prolonged remission, required fewer UV sessions and lower UV doses to attain remission, and had similar response rates compared to PUVA alone.^31-33^ Given the promising results of Re-UV in other conditions, the combination may be a welcome addition to the armamentarium for steroid refractory CHD.

The strengths of this study include its real-world setting and interesting results of statistically significant differences in clinical outcomes and adverse effect profiles between cohorts. However, the study was limited by its relatively small sample size and retrospective design. Treatment duration was only 16 weeks, and results of further follow-up were not reported. A potential challenge of the LDA-UVB treatment was that it required participants to return to the dermatology clinic three times weekly for their phototherapy treatment. This treatment burden may partially explain why our study did not find a significant difference in DLQI scores after treatment between cohorts. Larger prospective studies that compare low dose oral alitretinoin with NB-UVB therapy to high dose oral alitretinoin, NB-UVB therapy alone, or oral placebo with NB-UVB therapy are needed to further evaluate the effects of Re-UV in CHD and to test for differences in cumulative UV exposure.

In conclusion, low dose oral alitretinoin (10 mg) combined with NB-UVB three times weekly provided higher skin clearance rates and fewer adverse effects compared to high dose oral alitretinoin (30 mg daily) in patients with CHD refractory to topical corticosteroids. To the best of our knowledge, this is the first investigation of Re-UV combination therapy in CHD refractory to topical corticosteroid therapy. Current guidelines for the treatment of hand dermatitis do not mention Re-UV therapy.^1,5,35,36^ A recent Cochrane Review of all therapies for hand dermatitis did not find a single study that investigated Re-UV therapy.^23^ A search of the online databases OVID Medline 1946 to present, Embase 1974 to present, the Cochrane Central Register of Controlled Trials (CENTRAL), and the Web of Science Core Collection 1976 to present did not retrieve any proposals or completed studies that assessed Re-UV in CHD.
